# Identification of genes that regulate multiple cellular processes/responses in the context of lipotoxicity to hepatoma cells

**DOI:** 10.1186/1471-2164-8-364

**Published:** 2007-10-09

**Authors:** Shireesh Srivastava, Zheng Li, Xuerui Yang, Matthew Yedwabnick, Stephen Shaw, Christina Chan

**Affiliations:** 1 Department of Chemical Engineering and Material Science, Michigan State University, East Lansing, MI 48824, USA; 2 Department of Biochemistry and Molecular Biology, Michigan State University, East Lansing, MI 48824, USA; 3 Department of Computer Science and Engineering, Michigan State University, East Lansing, MI 48824, USA; 4 National Institute on Alcohol Abuse and Alcoholism (NIAAA/NIH), 5625 Fishers Lane, Rockville, MD, 20851, USA; 5 Biomedical Engineering Department, Boston University, Boston, MA 02215, USA

## Abstract

**Background:**

In order to devise efficient treatments for complex, multi-factorial diseases, it is important to identify the genes which regulate multiple cellular processes. Exposure to elevated levels of free fatty acids (FFAs) and tumor necrosis factor alpha (TNF-α) alters multiple cellular processes, causing lipotoxicity. Intracellular lipid accumulation has been shown to reduce the lipotoxicity of saturated FFA. We hypothesized that the genes which simultaneously regulate lipid accumulation as well as cytotoxicity may provide better targets to counter lipotoxicity of saturated FFA.

**Results:**

As a model system to test this hypothesis, human hepatoblastoma cells (HepG2) were exposed to elevated physiological levels of FFAs and TNF-α. Triglyceride (TG) accumulation, toxicity and the genomic responses to the treatments were measured. Here, we present a framework to identify such genes in the context of lipotoxicity. The aim of the current study is to identify the genes that could be altered to *treat *or ameliorate the cellular responses affected by a complex disease rather than to identify the causal genes. Genes that regulate the TG accumulation, cytotoxicity or both were identified by a modified genetic algorithm partial least squares (GA/PLS) analysis. The analyses identified NADH dehydrogenase and mitogen activated protein kinases (MAPKs) as important regulators of both cytotoxicity and lipid accumulation in response to FFA and TNF-α exposure. In agreement with the predictions, inhibiting NADH dehydrogenase and c-Jun N-terminal kinase (JNK) reduced cytotoxicity significantly and increased intracellular TG accumulation. Inhibiting another MAPK pathway, the extracellular signal regulated kinase (ERK), on the other hand, improved the cytotoxicity without changing TG accumulation. Much greater reduction in the toxicity was observed upon inhibiting the NADH dehydrogenase and MAPK (which were identified by the dual-response analysis), than for the stearoyl-CoA desaturase (SCD) activation (which was identified for the TG-alone analysis).

**Conclusion:**

These results demonstrate the applicability of GA/PLS in identifying the genes that regulate multiple cellular responses of interest and that genes regulating multiple cellular responses may be better candidates for countering complex diseases.

## Background

Many human diseases result from alterations in multiple cellular processes. Therefore, targeting an individual process or response may not be sufficient to combat the progression of such diseases. For efficient treatment of these diseases, it is imperative to identify those cellular targets that control multiple functions of interest. For example, prolonged exposure of cells to elevated levels of FFAs can alter multiple cellular processes such as reactive oxygen species (ROS) production [1,2] and cause mitochondrial [3], endoplasmic reticular [4,5] and lysosomal [6] dysfunction and activate a variety of signaling pathways, e.g,, protein kinase C (PKC) [7] and caspases [8]. The resultant effect of these disturbances is lipotoxicity, that is, cell death due to exposure to elevated lipid levels.

Many recent studies have identified that saturated FFAs cause greater lipotoxicity than unsaturated FFAs. It has been shown that channeling the saturated fatty acids to TG reduces their cytotoxicity [9]. Thus, the genes that regulate TG synthesis from saturated FFAs could be useful targets to combat the lipotoxic action of saturated FAs. We hypothesized that the genes which affect lipid synthesis as well as lipotoxicity could be better targets than those regulating TG synthesis alone. To test this hypothesis, we studied the cytotoxicity and TG accumulation in response to various types of FFAs and concentrations of TNF-α. The global gene expressions were obtained by cDNA microarray analyses.

It is important to identify associations between phenotype and genotype in order to understand and create models of disease mechanisms. These associations can help to identify critical pathways, genes and proteins that regulate biological processes, and in turn provide better drug targets to regulate diseases. A challenge in analyzing microarray data is the high dimensionality. The number of variables (genes) is usually on the order of thousands, while the number of observations (biological samples) is usually on the order of ten or twenty. Also, since there is a high degree of collinearity in the gene expression data, it is unfeasible to apply conventional statistical approaches to gene expression data [10]. Therefore, various approaches have been developed to reduce the dimensionality and mine information from microarray data. A common approach to reduce the dimensionality is principal component analysis (PCA). However, extracting the PCs from gene expression data does not require nor accommodate phenotypic profiles. Thus, PCA is not optimal for phenotype prediction [10]. Clustering has been applied to identify co-expressed genes which are often hypothesized to be co-regulated. The gene clusters that are identified offer insights into the biological processes [11]. However, clustering techniques cannot quantitatively link the genes to the phenotype profiles to provide predictions. Regression analysis is one approach to quantitatively link genes to phenotype profiles. However, the number of samples required should exceed the number of variables in the analysis, which is typically not the case. To overcome these problems, partial least squares analysis (PLS) was chosen for this work. PLS has proven to be a useful approach for analyzing data with strong collinearity [10]. It circumvents the typical problems associated with highly correlated and collinear data by projecting the data onto a new set of fewer unobserved, independent latent variables. PLS has been applied widely to microarray data for prediction and classifications [10,12,13]. To select the genes for prediction, we applied an approach based upon genetic algorithms (GA). Genetic algorithm coupled partial least squares (GA/PLS) has been applied successfully to identify the genes relevant to individual cellular functions, such as urea production and triglyceride synthesis [12]. While GA/PLS has hitherto been applied to identify genes relevant to individual responses, we hypothesized that a similar approach could be developed to identify genes that regulate multiple cellular responses. By regulating multiple processes, the genes that are identified could serve as useful targets to treat multi-factorial diseases that affect more than one cellular process or response.

Combining GA with PLS, however, typically results in multiple distinct PLS regression models from different subsets of genes that predict the phenotype profile with similar accuracies [12]. Therefore, to address this, we ran the GA/PLS algorithm multiple times to search from a larger number of possible combinations of genes and counted the frequency of appearance of each gene in the solution space. Increasing the number of runs increased the sample size or the number of genes drawn from the solution space [14]. The probabilistic nature of this approach improved the robustness and accuracy of the phenotype prediction by the GA/PLS model. Genes with high frequencies were selected into the final set of genes which were used to predict the phenotype.

The effect of perturbing genes identified to regulate both lipid accumulation and toxicity were compared to those that were identified for TG or LDH-alone. Many genes with known roles in lipid metabolism (e.g. stearoyl-CoA desaturase (SCD) and inositol polyphosphate phosphatase-like 1 (INPPL1)) were identified by the GA/PLS analysis for TG accumulation, attesting to the applicability of this approach. Then, the dual-response GA/PLS analysis was applied to identify the genes that regulate both TG accumulation and cytotoxicity. In addition to the genes with known roles in the regulation of lipid metabolism and cell physiology, this analysis identified NADH dehydrogenases and MAPKs as important regulators of both lipid accumulation and cell death. While NADH dehydrogenases and MAPKs have been shown to play roles in cell death caused by other insults, this analysis suggested that they affect not only cell death in response to FFAs, but also lipid accumulation. The predicted roles of NADH dehydrogenases and MAPKs were verified experimentally by employing inhibitors specific to them. Inhibiting NADH dehydrogenase and JNK significantly reduced the cytotoxicity and increased the intracellular TG accumulation, while inhibiting ERK reduced the toxicity without changing the lipid accumulation.

This study demonstrates the applicability of the dual-response GA/PLS analysis to identify genes which affect diverse cellular processes, and that incorporating more information provides better targets to control cellular responses. It must be pointed out that although the method was applied to identify the genes regulating two responses in the current study, the method is capable of handling more than two responses for multi-factorial diseases.

## Methods

### Materials

HepG2/C3A cells and Fetal Bovine Serum (FBS) were purchased from American Type Culture Collection (ATCC, Manassas, VA). Dulbecco's modified Eagle's medium with high glucose and no pyruvate (DMEM), Penicillin-Streptomycin (P/S), phosphate buffered saline (PBS, pH 7.4) and Trizol reagent were purchased from Invitrogen (Carlsbad, CA). Fatty acid free bovine serum albumin (BSA) was purchased from MP Biomedicals (Chillicothe, OH). Sodium salts of all the fatty acids (palmitate, oleate and linoleate) were purchased from Sigma Aldrich chemical company (St. Louis, MO). 6-carboxy-2',7'-dichlorodihydrofluorescein diacetate, di(acetoxymethyl ester) (DCFDA dye) was obtained from Molecular Probes (Eugene, OR). Recombinant human TNF-α was from Peprotech (Rocky Hill, NJ).

### Cell Culture

HepG2/C3A cells were cultured in 2 ml of medium containing DMEM supplemented with 10% fetal bovine serum (FBS) and 2% Penicillin-streptomycin (P/S). Cells were incubated at 37°C and in 10% CO_2 _atmosphere. Upon reaching confluence, cells were treated with either the control media (HepG2 medium or 4% BSA) or the FFA medium containing 0.7 mM palmitate, oleate or linoleate, or the FFA-TNF-α medium. The fatty acids were chosen because they are the most prevalent in the class of saturated (palmitate), monounsaturated (oleate) and polyunsaturated (linoleate) fatty acids in the plasma. The concentration of fatty acids chosen (0.7 mM) is commonly found in conditions of obesity. The FFAs were dissolved in 4% fatty acid-free BSA. Therefore, in addition to the HepG2 medium control, 4% fatty acid-free BSA in HepG2 medium was used as another control. TNF-α was added from a 100 μg/ml stock in deionized water to make the desired final concentrations of either 20 or 100 ng/ml. The experimental design is summarized in Table 1. The numbers at the end of the medium name represent the concentration of TNF-α in ng/ml, e.g., the culture medium containing 0.7 mM palmitate without TNF-α is represented as Palm0 (or P-0); 0.7 mM palmitate with 20 ng/ml TNF-α is represented as Palm20 (or P-20); 0.7 mM palmitate with 100 ng/ml TNF-α is represented as Palm100 (or P-100).

**Table 1:** The cytotoxicity of various treatments

**Medium**	**TNF (ng/ml)**	**LDH Released (%)**	
		**Mean**	**SD**
HG2	0	1.12	0.68
HG2	20	1.64	1.06
HG2	100	1.58	0.69
BSA	0	1.52	0.85
BSA	20	2.23	1.42
BSA	100	1.95	0.92
Palm	0	4.67 *	1.22
Palm	20	8.87 * ^#^	2.3
Palm	100	9.82 * ^#^	2.13
Oleate	0	1.34	0.8
Oleate	20	1.35	0.48
Oleate	100	1.43	0.35
Linoleate	0	1.23	0.46
Linoleate	20	1.82	0.39
Linoleate	100	1.25	0.1

*, significantly different than the control (HG2 – HepG2 cells exposed to culture medium, p < 0.01). #, significantly different than the Palmitate condition with no TNF-α (p < 0.05).

### Cytotoxicity measurement

The cytotoxicity was measured as the fraction of lactate dehydrogenase (LDH) released into the medium. Cells were cultured in different media for 24 h and the supernatants were collected. Cells were washed with phosphate buffered saline (PBS) and lyzed in 1% triton-X-100 in PBS. Cell lysates were collected, vortexed for 15 seconds and centrifuged at 7000 rpm for 5 minutes. Cytotoxicity detection kit (Roche Applied Science, Indianapolis, IN) was used to measure the percentage of LDH released, which was calculated as shown below

%LDHrelease=LDH(medium)LDH(total)×100     (1)
 MathType@MTEF@5@5@+=feaafiart1ev1aaatCvAUfKttLearuWrP9MDH5MBPbIqV92AaeXatLxBI9gBaebbnrfifHhDYfgasaacH8akY=wiFfYdH8Gipec8Eeeu0xXdbba9frFj0=OqFfea0dXdd9vqai=hGuQ8kuc9pgc9s8qqaq=dirpe0xb9q8qiLsFr0=vr0=vr0dc8meaabaqaciaacaGaaeqabaqabeGadaaakeaacqGGLaqjcqWGmbatcqWGebarcqWGibascqWGYbGCcqWGLbqzcqWGSbaBcqWGLbqzcqWGHbqycqWGZbWCcqWGLbqzcqGH9aqpdaWcaaqaaiabdYeamjabdseaejabdIeaijabcIcaOiabd2gaTjabdwgaLjabdsgaKjabdMgaPjabdwha1jabd2gaTjabcMcaPaqaaiabdYeamjabdseaejabdIeaijabcIcaOiabdsha0jabd+gaVjabdsha0jabdggaHjabdYgaSjabcMcaPaaacqGHxdaTcqaIXaqmcqaIWaamcqaIWaamaaa@596A@

For experiments with inhibition of NADH dehydrogenases, the cells were pre-treated with 0.2–1.0 μM rotenone in the culture medium for 30 min, followed by exposure to 0.7 mM palmitate for 24 h without any inhibitor. The LDH released after the 24 h exposure was measured and normalized to the triton-lyzed values, as described above.

For experiments with the MAPK-inhibitors, stock solutions of these inhibitors, were prepared in DMSO and diluted to desired concentrations in palmitate medium. Cells were treated with the palmitate medium containing the desired concentrations of the MAPK inhibitors. Under no condition was the concentration of the resulting DMSO greater than 0.2% v/v. At these concentrations, DMSO does not affect the cytotoxicity or the TG levels.

### Measurement of intracellular TG levels

The cells were pretreated for 30 minutes with 0–1 μM rotenone, and then exposed to 0.7 mM palmitate or oleate. In the experiments with MAPK inhibitors, cells were treated with the desired concentrations of the inhibitor dissolved in the FFA medium. After 24 h of exposure to the FFAs, the cells were washed thrice with PBS and lyzed with 1% triton in PBS. The lysates were centrifuged at 10000 g for 2 min, and the concentration of TG in the supernatant was measured by an enzymatic assay kit from Stanbio, according to manufacturer's instructions. This method measures the amount of glycerol released by the enzymatic hydrolysis of the TG. To correct for the free glycerol in the cells, the concentration of free glycerol in the cells were measured by free glycerol assay from Sigma, and subtracted from the glycerol values of the TG assay, to give actual TG values. These values were normalized to total protein in the extract, measured with the bicinchoninic acid (BCA) method (Pierce Chemicals).

### Measurement of caspase-3 activity

Caspase-3 activity was measured using a commercially available colorimetric assay (Biovision) according to manufacturer's instructions. The cells were treated with the control medium or 0.7 mM palmitate for 24 h, washed once with PBS and lyzed using the available lysis buffer. The activity in the lysate was measured and normalized to the total protein, measured by bicinchoninic acid (BCA) assay (Pierce Chemicals).

### Measurement of intracellular ATP levels

Intracellular ATP levels were measured using an ATP determination kit (Invitrogen) according to manufacturer's instructions. The cells were pre-treated with 0, 0.25, 0.5, or 1.0 μM rotenone in the culture medium for 30 min, followed by exposure to 0.7 mM palmitate for 24 h without any inhibitor. The cells were then washed twice with PBS and lyzed using the available lysis buffer. The ATP levels in the lysate were measured using a luminometer and normalized to the total protein, measured by bicinchoninic acid (BCA) assay (Pierce Chemicals).

### RNA isolation

Cells were cultured in 10 cm tissue culture plates until confluence and then exposed to different treatments for 24 h. Cells were then washed twice with ice-cold phosphate buffered saline (PBS). PBS was then aspirated and 10 ml trizol reagent was added. After leaving the cells in trizol for 5–10 minutes, the cell lysate was transferred to 15 ml conical tubes and vortexed. Meanwhile, phase-lock gel (heavy) in 15 ml tubes was centrifuged at 1000 g for 10 min. Trizol cell lysate was added to tubes containing phase-lock gel. 3 ml of chloroform was added to the tubes and mixed. The tubes were then centrifuged at 4000 g for 30 minutes to separate the aqueous and organic phases, with the phase-lock gel forming a central layer. The aqueous phase was poured off into fresh 15 ml conical tubes. 5 ml of isopropyl alcohol was then added to the tubes containing the aqueous phase and tubes were mixed gently by inversion. Tubes were then centrifuged at 4000 g for 20 minutes. RNA forms a pellet at the bottom. The supernatant liquid is decanted and the pellets were washed thrice with ice-cold 75% ethanol. Ethanol was then carefully removed and the pellet suspended in 1 ml of water and the solution was transferred to 1.5 ml microcentrifuge tubes. For LiCl precipitation, 0.5 ml of 7.5 M LiCl was added to the tubes and vortexed. The tubes were then kept overnight at -20C. The next morning, tubes were centrifuged at 13000 g for 45 min. The pellet was washed thrice with ice-cold 75% ethanol. Finally, all the ethanol was removed and the pellet suspended in 100 ul RNAase-free water.

### Generation of cDNAs, hybridization and microarray analyses

These steps were performed at the Van Andel Research Institute. The protocols used are available online [15]. Briefly, labeled cDNAs were generated with Reverse Transcriptase (RT) reaction using Cy3 or Cy5 -labeled dCTP and low-dCTP dNTP mix. After generating labeled cDNA, the template was degraded using Rnase and the cDNA further purified using QIAquick columns (Qiagen, Valencia, CA). Hybridization reactions were performed in a 50C water bath for 16 h, following which the microarrays were washed and read.

There were two biological replicates for each condition and each replicate was measured with the Cy3 and Cy5 dyes, i.e. there were two technical replicates/color swaps for each biological replicate. Color swaps indicate the arrays in which the cDNA from the treated sample was labeled with Cy3 dye and the cDNA from the control sample was labeled with the Cy5 dye.

### RT-PCR

Total RNA was extracted from cells with an RNeasy mini kit (Qiagen) and depleted of contaminating DNA with RNase-free DNase (Qiagen). Equal amounts of total RNA (1 μg) were reverse-transcribed using an iScript cDNA synthesis kit (Bio-Rad). The first-strand cDNA was used as a template. The primers used for quantitative RT-PCR analyses of human SCD (5'-TGAGAAACTGGTGATGTTCCA-3' and 5'-CCACAGCATATCGCAAGAAA-3'), human INPPL1 (5'-GCTGGGTGTTGTGTTTGAGA-3' and 5'-TCCTGCTTGGAGTGCTTGT-3') and human GAPDH (5'-AACTTTGGTATCGTGGAAGGA-3' and 5'-CAGTAGAGGCAGGGATGATGT-3') were synthesized by Operon Biotechnologies, Inc. RT-PCR was performed in 25-μl reactions using 1/10 of the cDNA obtained from the reverse transcription, 0.2 μM each primer, 1X SYBR green supermix from Bio-RAD, and an annealing temperature of 60°C for 40 cycles. Each sample was assayed in three independent RT reactions and triplicate reactions each and normalized to GAPDH expression. Negative controls included the absence of enzyme in the RT reaction and absence of template during PCR. The cycle threshold (CT) values corresponding to the PCR cycle number at which fluorescence emission in real time reaches a threshold above the base-line emission were determined using MyIQ™ Real-Time PCR Detection System.

### Western Blots

For the western blots, cells were treated for 24 h with FFAs or BSA, then thrice washed with 1 ml ice-cold PBS per well. The cells were then scraped in 200 ul ice-cold Cell Lytic M lysis buffer from Sigma and sonicated for 20 seconds, followed by centrifugation at 5000 g for 2 minutes at 4°C. The supernatants were added to 3X SDS sample buffer from New England Biolabs and heated at 95°C for 10 minutes, followed by cooling on ice for 1 minute and centrifugation at 5000 g for 2 minute. Samples were then loaded onto 7.5% Tris-HCL gel and electrophoresed at 110 V for 90 min. Samples were blotted onto nitrocellulose membrane at 110 V for 60 min. The membrane was blocked with 5% non-fat fry milk (Bio-Rad) in tris buffered saline containing 0.5% tween-20 (TBS-T). The membranes were washed thrice with TBS-T, followed by exposure to AMPKα antibody (Sigma-Aldrich), SCD antibody (Cell Signaling Technologies), or p-AMPKα Thr172 antibody (Cell Signaling Technologies) at 1:1000 in TBT-T for 16 h at 4°C. Membranes were washed thrice with TBS-T and exposed to the secondary antibody (HRP conjugated rabbit anti-IgG) for 1 h at room temperature. The blots were washed thrice with TBS-T, developed using Supersignal West Femto Substrate (Pierce Chemicals) and imaged using a Bio-Rad imager.

### Single and dual response GA/PLS analysis

Metabolic functions are regulated in part by the enzymes catalyzing the reactions, which in turn are determined partly by their gene expression levels. Therefore, we hypothesized that the metabolic functions can be predicted from the expression level of a subset of genes that are associated with the metabolic function. We approximated the relation between the metabolic function and expression level of this subset of genes with a log-linear model:

Met(treatment)Met(control)=∏i=1n(Gene(treatment)iGene(control)i)C(i)     (2)
 MathType@MTEF@5@5@+=feaafiart1ev1aaatCvAUfKttLearuWrP9MDH5MBPbIqV92AaeXatLxBI9gBaebbnrfifHhDYfgasaacH8akY=wiFfYdH8Gipec8Eeeu0xXdbba9frFj0=OqFfea0dXdd9vqai=hGuQ8kuc9pgc9s8qqaq=dirpe0xb9q8qiLsFr0=vr0=vr0dc8meaabaqaciaacaGaaeqabaqabeGadaaakeaadaWcaaqaaiabd2eanjabdwgaLjabdsha0jabcIcaOiabdsha0jabdkhaYjabdwgaLjabdggaHjabdsha0jabd2gaTjabdwgaLjabd6gaUjabdsha0jabcMcaPaqaaiabd2eanjabdwgaLjabdsha0jabcIcaOiabdogaJjabd+gaVjabd6gaUjabdsha0jabdkhaYjabd+gaVjabdYgaSjabcMcaPaaacqGH9aqpdaqeWbqaaiabcIcaOmaalaaabaGaem4raCKaemyzauMaemOBa4MaemyzauMaeiikaGIaemiDaqNaemOCaiNaemyzauMaemyyaeMaemiDaqNaemyBa0MaemyzauMaemOBa4MaemiDaqNaeiykaKYaaSbaaSqaaiabdMgaPbqabaaakeaacqWGhbWrcqWGLbqzcqWGUbGBcqWGLbqzcqGGOaakcqWGJbWycqWGVbWBcqWGUbGBcqWG0baDcqWGYbGCcqWGVbWBcqWGSbaBcqGGPaqkdaWgaaWcbaGaemyAaKgabeaaaaGccqGGPaqkdaahaaqabeaacqWGdbWqcqGGOaakcqWGPbqAcqGGPaqkaaaaleaacqWGPbqAcqGH9aqpcqaIXaqmaeaacqWGUbGBa0Gaey4dIunaaaa@8319@

where *Met(treatment) *and *Met(control) *are the metabolic function for the treated and control cultures, respectively; *Gene(treatment)*_*i *_and *Gene(control)*_*i *_are the expression level of gene *i *for the treated and control cultures, respectively.

Denoting Y as log⁡2(Met(treatment)Met(control))
 MathType@MTEF@5@5@+=feaafiart1ev1aaatCvAUfKttLearuWrP9MDH5MBPbIqV92AaeXatLxBI9gBaebbnrfifHhDYfgasaacH8akY=wiFfYdH8Gipec8Eeeu0xXdbba9frFj0=OqFfea0dXdd9vqai=hGuQ8kuc9pgc9s8qqaq=dirpe0xb9q8qiLsFr0=vr0=vr0dc8meaabaqaciaacaGaaeqabaqabeGadaaakeaacyGGSbaBcqGGVbWBcqGGNbWzdaWgaaWcbaGaeGOmaidabeaakiabcIcaOmaalaaabaGaemyta0KaemyzauMaemiDaqNaeiikaGIaemiDaqNaemOCaiNaemyzauMaemyyaeMaemiDaqNaemyBa0MaemyzauMaemOBa4MaemiDaqNaeiykaKcabaGaemyta0KaemyzauMaemiDaqNaeiikaGIaem4yamMaem4Ba8MaemOBa4MaemiDaqNaemOCaiNaem4Ba8MaemiBaWMaeiykaKcaaiabcMcaPaaa@5520@ and X_i _as log⁡2(Gene(treatment)iGene(control)i)
 MathType@MTEF@5@5@+=feaafiart1ev1aaatCvAUfKttLearuWrP9MDH5MBPbIqV92AaeXatLxBI9gBaebbnrfifHhDYfgasaacH8akY=wiFfYdH8Gipec8Eeeu0xXdbba9frFj0=OqFfea0dXdd9vqai=hGuQ8kuc9pgc9s8qqaq=dirpe0xb9q8qiLsFr0=vr0=vr0dc8meaabaqaciaacaGaaeqabaqabeGadaaakeaacyGGSbaBcqGGVbWBcqGGNbWzdaWgaaWcbaGaeGOmaidabeaakiabcIcaOmaalaaabaGaem4raCKaemyzauMaemOBa4MaemyzauMaeiikaGIaemiDaqNaemOCaiNaemyzauMaemyyaeMaemiDaqNaemyBa0MaemyzauMaemOBa4MaemiDaqNaeiykaKYaaSbaaSqaaiabdMgaPbqabaaakeaacqWGhbWrcqWGLbqzcqWGUbGBcqWGLbqzcqGGOaakcqWGJbWycqWGVbWBcqWGUbGBcqWG0baDcqWGYbGCcqWGVbWBcqWGSbaBcqGGPaqkdaWgaaWcbaGaemyAaKgabeaaaaGccqGGPaqkaaa@5AB8@, equation (2) is transformed to:

Y=∑i=1nC(i)Xi     (3)
 MathType@MTEF@5@5@+=feaafiart1ev1aaatCvAUfKttLearuWrP9MDH5MBPbIqV92AaeXatLxBI9gBaebbnrfifHhDYfgasaacH8akY=wiFfYdH8Gipec8Eeeu0xXdbba9frFj0=OqFfea0dXdd9vqai=hGuQ8kuc9pgc9s8qqaq=dirpe0xb9q8qiLsFr0=vr0=vr0dc8meaabaqaciaacaGaaeqabaqabeGadaaakeaacqWGzbqwcqGH9aqpdaaeWbqaaiabdoeadjabcIcaOiabdMgaPjabcMcaPaWcbaGaemyAaKMaeyypa0JaeGymaedabaGaemOBa4ganiabggHiLdGccqWGybawdaWgaaWcbaGaemyAaKgabeaaaaa@3CCB@

Since DNA microarray data are typically measured with respect to a reference level, we applied a log-linear model, which works well when the data are presented as relative levels. Furthermore, a log-linear model allowed some of the nonlinear relationships between metabolic function and gene expression to be captured. Log-linear models have been applied to approximate nonlinear processes in biochemical systems [16,17]. In this study the coefficients *C(i) *in equation (3) were determined by PLS analysis. C(i)s are the regression coefficients and they provide the weights for the genes in the prediction model (equation 3). Thus, C(i) indicates the relative importance of gene (i) in predicting the metabolic function. The genes, *Gene*_*i*_, were selected by GA/PLS as described by [12]. Briefly, multiple subsets of genes that provide similar prediction of the metabolic functions using equation (3) were identified and the frequency of appearance of each gene in these subsets was counted. Those genes with high frequencies were deemed important genes to the metabolic function.

To identify genes relevant to multiple cellular functions, GA/PLS [12] was modified by expanding *Y *to be a vector of multiple metabolites. A linear prediction model of partial least squares analysis (PLS) was used to estimate the regression parameters. We used PLS to map the levels of gene expression (*X*) to a metabolic function (*Y*), to gain an understanding of the interplay between a cellular function and the gene expression profile. The PLS algorithm determined, based upon a nonlinear iterative partial least squares (NIPALS) approach [18], a set of orthogonal projection axes *W*, henceforth called PLS-weights, and sample projections *T*. For direct projection of the samples, W* = (*W*(PT*W)^-1^) was used:

*T *= XW*     (4)

Then, regression coefficients *β *in equation (5) were obtained by regressing *Y *onto the sample projections *T*:

*Y *= *Tβ*     (5)

With *a *PLS factors, the PLS model is:

Y^=XWa∗β=Taβ=∑j=1atjβj     (6)
 MathType@MTEF@5@5@+=feaafiart1ev1aaatCvAUfKttLearuWrP9MDH5MBPbIqV92AaeXatLxBI9gBaebbnrfifHhDYfgasaacH8akY=wiFfYdH8Gipec8Eeeu0xXdbba9frFj0=OqFfea0dXdd9vqai=hGuQ8kuc9pgc9s8qqaq=dirpe0xb9q8qiLsFr0=vr0=vr0dc8meaabaqaciaacaGaaeqabaqabeGadaaakeaacuWGzbqwgaqcaiabg2da9iabdIfayjabdEfaxnaaDaaaleaacqWGHbqyaeaacqGHxiIkaaacciGccqWFYoGycqGH9aqpcqWGubavdaWgaaWcbaGaemyyaegabeaakiab=j7aIjabg2da9maaqahabaGaemiDaq3aaSbaaSqaaiabdQgaQbqabaGccqWFYoGydaWgaaWcbaGaemOAaOgabeaaaeaacqWGQbGAcqGH9aqpcqaIXaqmaeaacqWGHbqya0GaeyyeIuoaaaa@48DE@

β=(TaTT)−1TaTY     (7)
 MathType@MTEF@5@5@+=feaafiart1ev1aaatCvAUfKttLearuWrP9MDH5MBPbIqV92AaeXatLxBI9gBaebbnrfifHhDYfgasaacH8akY=wiFfYdH8Gipec8Eeeu0xXdbba9frFj0=OqFfea0dXdd9vqai=hGuQ8kuc9pgc9s8qqaq=dirpe0xb9q8qiLsFr0=vr0=vr0dc8meaabaqaciaacaGaaeqabaqabeGadaaakeaaiiGacqWFYoGycqGH9aqpdaqadaqaaiabdsfaunaaDaaaleaacqWGHbqyaeaacqWGubavaaGccqWGubavaiaawIcacaGLPaaadaahaaWcbeqaaiabgkHiTiabigdaXaaakiabdsfaunaaDaaaleaacqWGHbqyaeaacqWGubavaaGccqWGzbqwaaa@3D2B@

The genes identified to be important to multiple metabolites (*Y*) were selected with a modified GA/PLS. Compared to the single metabolite analysis, the genes were selected so that the overall prediction accuracy for all the metabolites, e.g. [*Y1*, *Y2*,...] was maximized. GA was applied to search for this maximum. GA begins with an initial, randomly selected population. A population is a collection of different individuals, i.e., a set of different strings (e.g., genes) of the same length. Each individual in the population is a potential solution to the optimization problem and its fitness to the optimization problem is evaluated by a fitness function (equation 8). An individual is a string of length *N *with the first *N-1 *values of the string being a binary with value 1/0 representing the inclusion or exclusion of a gene in the PLS model and the *N*^*th *^value representing the number of latent variables in the PLS model.

Each individual in the population is evaluated with a fitness function to determine how well they fit or improved the optimization problem. The optimization problem in our case is to find a subset of genes that can be used to construct a PLS model to predict metabolic functions with minimal prediction error and number of latent variables. The fitness function was modified to include more metabolites shown in equation (8), where

fitness=1∑i∑j(yi,j−y^i,j)2+LVw     (8)
 MathType@MTEF@5@5@+=feaafiart1ev1aaatCvAUfKttLearuWrP9MDH5MBPbIqV92AaeXatLxBI9gBaebbnrfifHhDYfgasaacH8akY=wiFfYdH8Gipec8Eeeu0xXdbba9frFj0=OqFfea0dXdd9vqai=hGuQ8kuc9pgc9s8qqaq=dirpe0xb9q8qiLsFr0=vr0=vr0dc8meaabaqaciaacaGaaeqabaqabeGadaaakeaacqWGMbGzcqWGPbqAcqWG0baDcqWGUbGBcqWGLbqzcqWGZbWCcqWGZbWCcqGH9aqpdaWcaaqaaiabigdaXaqaamaaqafabaWaaabuaeaacqGGOaakcqWG5bqEdaWgaaWcbaGaemyAaKMaeiilaWIaemOAaOgabeaakiabgkHiTiqbdMha5zaajaWaaSbaaSqaaiabdMgaPjabcYcaSiabdQgaQbqabaGccqGGPaqkdaahaaWcbeqaaiabikdaYaaaaeaacqWGQbGAaeqaniabggHiLdaaleaacqWGPbqAaeqaniabggHiLdGccqGHRaWkcqWGmbatcqWGwbGvdaahaaWcbeqaaiabdEha3baaaaaaaa@52A2@

*y*_*i, j *_is the *i*^*th *^measured response for the *j*^*th *^treatment condition, y^i,j
 MathType@MTEF@5@5@+=feaafiart1ev1aaatCvAUfKttLearuWrP9MDH5MBPbIqV92AaeXatLxBI9gBaebbnrfifHhDYfgasaacH8akY=wiFfYdH8Gipec8Eeeu0xXdbba9frFj0=OqFfea0dXdd9vqai=hGuQ8kuc9pgc9s8qqaq=dirpe0xb9q8qiLsFr0=vr0=vr0dc8meaabaqaciaacaGaaeqabaqabeGadaaakeaacuWG5bqEgaqcamaaBaaaleaacqWGPbqAcqGGSaalcqWGQbGAaeqaaaaa@31FB@ is the corresponding PLS predicted value, *LV *is the number of latent variables in the PLS model and *w *is a weighting factor to establish an optimal balance between prediction accuracy and the model size (number of PLS latent variables). A value of *w *= 0.3 was used here, as determined using the method described in [12].

The initial population is created randomly in a user specified bound of the *N *variables in the string. The population evolves over generation in three ways: reproduction, crossover and mutation. The process terminates when the objective function reaches its maximum or when the termination condition (e.g., maximum number of iterations) is satisfied.

GA can not guarantee a global optimum, thus GA/PLS selects different subsets of genes to predict the same cellular function given different initial populations. Therefore, as described in [12] we ran the GA/PLS model with different initial populations and counted the frequency of appearance of each gene in the multiple solutions. The initial population size ranged from 30 to 100 individuals and each individual contained a set of different genes. GA/PLS was run 14 times with different sizes of initial populations. A gene was included in the final subset if it was selected by the GA/PLS model in more than half of the runs. Therefore, the genes that appeared more than 8 times as a solution in the GA/PLS model were selected into the final gene subset. A web platform of the GA/PLS methods can be accessed at [19].

GA/PLS was used to determine a set of possible solutions rather than a single solution. With this method, multiple solutions of different subsets of genes gave similar prediction accuracy. We explored the solution space by selecting genes based upon their frequency of appearance in the multiple runs. In other words, the probability of significant features (important genes) appearing in the solution space was estimated based upon their frequency. The probabilistic nature of this method improved the robustness of the GA/PLS approach. Increasing the number of runs provided a larger sample size that was drawn from the solution space [20]. However, running GA/PLS is very time consuming with each run taking around 1 hour on a PC with Celeron CPU 2.4 GHZ and RAM 512 MB. Therefore, it is of interest to determine the minimum number of GA/PLS runs that would provide a set of genes that would not change significantly, i.e. a robust set of genes. To estimate the number of runs required, we evaluated the robustness of the results to the number of runs performed. We changed the number of total runs from 3, 6, 7, 12, 14, 20 to 24. The frequency with which each gene was selected in the different runs can be found in additional data file 1. The genes selected did vary with the number of runs. However, we observed that more than 92% of the 830 genes remained selected when the runs were increased to 14 and higher, suggesting that 14 runs were sufficient. This indicated that changing the total number of times the GA/PLS algorithm was run beyond 14 did not alter significantly the genes selected by GA/PLS, i.e., 14 runs were sufficient. Therefore, genes selected after 14 runs were used for further analysis and validation.

CHEMOMETRICS toolbox from MathWorks was used for implementing PLS and defining the fitness function. Genetic Algorithm Optimization Toolbox (GAOT) [21] was used for Genetic Algorithm implementation.

### Statistical analyses

Analysis of variance (ANOVA) was applied to compare the effects of treatment (e.g. FFA, TNF-α) and to determine whether a treatment had a significant effect. We applied two-way ANOVA to identify the genes that were affected by FFA, TNF-α or their interaction. The analysis was performed in MATLAB 6.3 using Stats Toolbox. A two step ANOVA analysis was performed to identify the genes that changed significantly due to FFA or TNF-α exposure. We identified a list of genes from the literature [20], that are relevant to palmitate-induced cytotoxicity and applied ANOVA with p < 0.05 to this list of genes (which we denote as ''supervised'' ANOVA). In addition, ANOVA analysis was applied to the entire list of genes with p < 0.01 (which we denote as ''unsupervised'' ANOVA). The two lists of genes were then combined into one list, eliminating any overlaps between the lists. The ESTs of hypothetical proteins and ORF of unknown functions were removed from the gene list. Using the supervised and unsupervised ANOVA tests, the expression level of 830 genes were found to be significant due to either TNF-α or FFA. The list of genes has been reported elsewhere in [20].

Significant treatment effects were statistically evaluated using ANOVA or t-test. For the cytotoxicity and TG accumulation measurements, two-way ANOVA followed by Bonferroni's post-hoc test was applied to identify significant differences. For the MAPK inhibition experiments an unpaired two-tailed t-test was performed. One-way ANOVA with Bonferroni post-hoc test was applied to compare the effect of treatments on caspase-3 activation.

## Results

### Lipid accumulation and cytotoxicity

**Figure 1:**
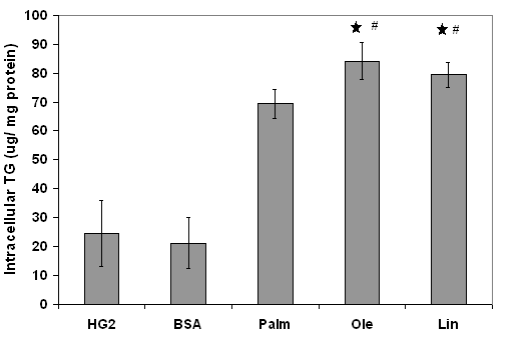
Intracellular accumulation of TG in response to various FFAs. Confluent HepG2 cells were exposed to various FFA for 24 h and the intracellular TG levels were measured enzymatically after cell lysis and normalized to the protein levels. Results presented as mean +/- s.d. of 3 independent experiments. , significantly different than control (p < 0.01), # significantly different from palmitate (p < 0.05).

Cells exposed to FFAs accumulated TG, which was quantified using an enzymatic assay. Among the FFA-treated cells, those treated with the saturated FFA, palmitate, had lower TG accumulation than the cells treated with the unsaturated FFAs, oleate and linoleate (Figure 1). No significant effect of TNF-α treatment on TG accumulation was observed (not shown), as reported also by others [22].

**Figure 2:**
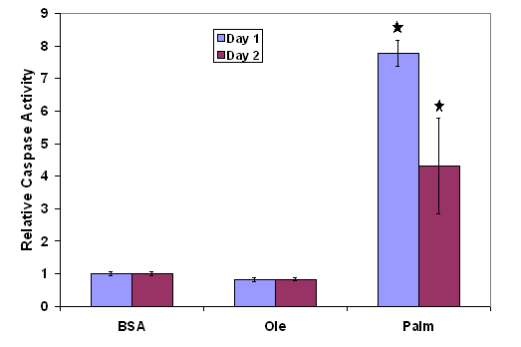
Effect of palmitate exposure on caspase-3 activation. Cells were treated with different FFAs for two days and the caspase-3 activity was measured using a fluorimetric assay. Caspase-3 activities relative to the control are presented. Data presented as mean +/- s.d. of 3 independent experiments. , significantly different from control (p < 0.01).

The cytotoxicity was measured after 24 h of treatment as the fraction of the total LDH activity released. The saturated fatty acid, palmitate, was found to be toxic to these cells and TNF-α exacerbated palmitate's toxicity, while the unsaturated fatty acids with or without TNF-α co-supplementation did not have a toxic effect (Table 1). Exposure to palmitate also increased the caspase-3 activity of the cells significantly, while oleate had no effect (Figure 2). For both LDH release as well as caspase-3 activation, it was observed that the effects of TNF-α were secondary to that of palmitate, i.e., TNF-α alone did not cause an increase in either LDH release or caspase-3 activation, but exacerbated the effects of palmitate on LDH release or caspase activation. Because palmitate was found to be the primary effector, most of the validation studies shown below were conducted with the palmitate samples only.

### Microarray analyses

To identify the genomic responses of the cells to the treatments, cDNA microarray analyses were performed. Two-way ANOVA followed by removal of ESTs identified that 830 genes were affected at a significance level of p < 0.01 by FFAs, TNF-α or their interaction. Specifically, the ESTs of hypothetical proteins and ORF of unknown functions were removed from the gene list after ANOVA analysis. Details of the pre-processing are provided in the materials and methods section. Further analyses were conducted on the selected genes to identify pathways and processes related to lipid accumulation and cytotoxicity. Since FFAs were the primary effectors of both lipid accumulation and cytotoxicity, while TNF-α alone had no significant effect on either the lipid accumulation or the toxicity, the validation studies were conducted on palmitate-treated cells only.

#### a. Identification of genes relevant to TG accumulation

**Figure 3:**
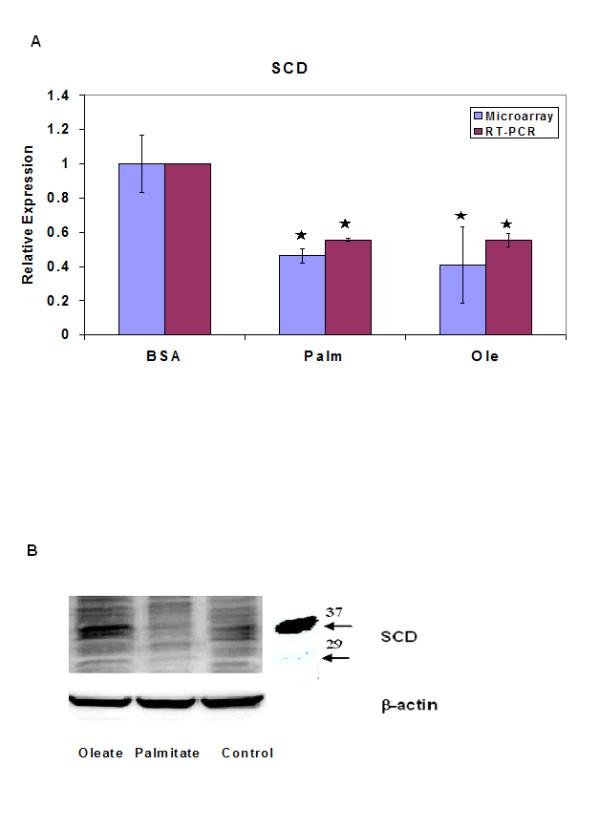
Effects of various FFAs on the expression of stearoyl-CoA desaturase (SCD). Cells were cultured in the presence of 0.7 mM palmitate or oleate for 24 h. (a) Effects on SCD transcript (mRNA) levels. RNA was extracted and the RT-PCR performed as explained in the materials and methods section. Results presented as transcript levels for the different conditions relative to control. Data shown as mean +/- s.d. of 3 independent experiments. , significantly different than control (p < 0.01). (b) SCD protein levels. After treating cells for 24 h with the FFAs, cells were lyzed and the protein levels of SCD were estimated using Western Blot. B = BSA medium, P = 0.7 mM, O = 0.7 mM oleate.

The GA/PLS analysis identified 88 genes among the 830 differentially expressed genes as relevant to TG accumulation. The complete list of selected genes can be found in additional file 2. Genes with the highest frequencies are listed in Table 2. Many genes with known roles in the regulation of lipid metabolism were identified. For example, SCD was identified as the most important gene in regulating TG accumulation. SCD catalyzes the unsaturation of stearoyl-CoA, the higher derivative of palmitate, to yield oleoyl-CoA. The activity of SCD has been shown to regulate hepatic TG synthesis in response to a lipogenic diet rich in saturated FFAs [23]. SCD increases TG synthesis by increasing the amounts of unsaturated FFA, a preferred substrate for monoacylglycerol acyltransferase (MGAT) which catalyzes the synthesis of diacylglycerol (DAG) [24]. Microarray results revealed that exposure to FFAs significantly reduced the transcript levels of SCD (Figure 3a). The results of the microarray analyses were confirmed by RT-PCR and good corroboration was obtained between the microarray and PCR results (Figure 3a). No significant effect of exposure to TNF-α was observed, either alone or in the presence of FFAs (not shown). Exposure to palmitate, but not to oleate, also caused a reduction in the protein levels (Figure 3b) of SCD. Reduced transcript levels, but no change in protein levels in cells exposed to unsaturated FFAs, suggests that these FFAs also reduced the rate of degradation of SCD so that the net protein levels are not changed significantly. Reduced SCD levels would hamper TG synthesis from palmitate, but not from unsaturated FFAs. The reduced TG synthesis from palmitate could exacerbate its toxicity. Increasing the levels of SCD has been shown to increase TG synthesis from palmitate and reduce its cytotoxicity [25].

**Table 2:** Top genes identified for TG (total 88 genes, see additional file 2 for a complete list)

**Accession Number**	**Gene Name**	**Freq**
AA457700	(gC) stearoyl-CoA desaturase (delta-9-desaturase) (SCD)	12
AA279072	(gK) inositol polyphosphate phosphatase-like 1 (INPPL1)	11
T81764	(gC) cell division cycle 27 (CDC27)	11
AA464195	(gC) protein phosphatase 1, regulatory (inhibitor) subunit 12C (PPP1R12C)	11
H16833	(gN) steroid-5-alpha-reductase, alpha polypeptide 1 (3-oxo-5 alpha-steroid delta 4-dehydrogenase alpha 1) (SRD5A1)	11
R33650	(gM) inner mitochondrial membrane peptidase 2 like (IMMP2L)	11

Another important regulator of TG synthesis identified by the GA/PLS analysis was INPPL1. The product of INPPL1 is the SH2-containing inositol 5'-phosphatase 2 (SHIP2), which is considered an important target in controlling obesity, type 2 diabetes and insulin resistance [26]. It has been shown that INPPL1-null mice are highly resistant to dietary obesity [27].

Thus, the genes identified by the GA/PLS analysis to be important in the regulation of TG accumulation have indeed been shown to play important roles in lipid accumulation. These results indicate the applicability of GA/PLS to identify genes relevant to a particular function of interest, e.g., TG accumulation.

#### b. Genes relevant to both TG accumulation and cytotoxicity identified by the dual-response GA/PLS

The dual-response GA/PLS (LDH + TG) identified 92 genes which may regulate both TG accumulation and cytotoxicity (see Table 3 for the genes with the highest frequencies and additional file 3 for a complete list). NADH dehydrogenase (ubiquinone) 1, alpha/beta sub complex, 1, 8 kDa (NDUFAB1) and mitogen-activated protein kinase kinase kinase kinase 2 (MAP4K2) were identified to have the highest frequency among the selected genes, suggesting that these genes affected lipid synthesis and cytotoxicity most significantly. NADH dehydrogenases formed the most prominent group of genes in the list, with 7 different NADH dehydrogenases selected. In accordance with this, the gene ontology tree machine (GOTM) analysis [28] also identified that the GO category of 'oxidoreductase activity, acting on NADH or NADPH, quinone or similar compound as acceptor' was significantly enriched (p < 0.01) in the selected list. These results indicated an important role of NADH dehydrogenases in regulating both cytotoxicity and TG accumulation.

**Table 3:** Top genes selected for TG+LDH by dual response GA/PLS (total 93 genes, see additional file 3 for a complete list)

**Accession Number**	**Name**	**Freq**
AA447569	(gN) NADH dehydrogenase (ubiquinone) 1, alpha/beta subcomplex, 1, 8 kDa (NDUFAB1)	12
R50953	(gC) mitogen-activated protein kinase kinase kinase kinase 2 (MAP4K2)	12
H23978	(gW) general transcription factor IIB (GTF2B)	12
T58773	(gN) inositol polyphosphate-5-phosphatase, 40 kDa (INPP5A)	11
AA464580	(gC) acetyl-Coenzyme A carboxylase alpha	11
AA431988	(gC) fatty acid amide hydrolase (FAAH)	11
AA463931	(gC) inositol 1,3,4-triphosphate 5/6 kinase (ITPK1)	11
N22904	(gC) 3-phosphoinositide dependent protein kinase-1 (PDPK1)	11
AA025112	(gC) BCL2/adenovirus E1B 19 kDa interacting protein 3-like	11
AA019459	(gC) protein tyrosine kinase 9 (PTK9)	11
AA064638	(gI) ubiquitin specific protease 7 (herpes virus-associated) (USP7)	11

**Figure 4:**
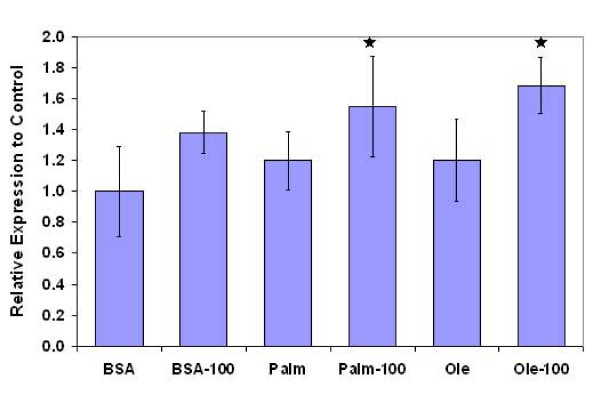
Effects of various FFAs on the expression of mitogen activated protein kinase kinase kinase kinase 2 (MAP4K2). Cells were cultured in the presence of 0.7 mM palmitate or oleate and 100 ng/ml TNF-α for 24 h. RNA was extracted and RT-PCR performed as explained in the materials and methods section. Results are presented as ratio of transcript levels with respect to control. Data presented as mean +/- s.d. of 3 independent experiments. , significantly different than control (p < 0.05). For the x-axis legends, the postscript '100' denotes the TNF-α concentration in ng/ml.

Another gene that had the highest frequency was the mitogen-activated protein kinase kinase kinase kinase 2 (MAP4K2). We tested the effects of various treatments on the expression of MAP4K2. Synergistic effects of fatty acids and TNF-α were observed on the expression of MAP4K2, i.e., either FFA or TNF-α alone increased the expression of MAP4K2 slightly but non-significantly. However, their interaction caused significant effect on MAP4K2 expression (Figure 4). MAP4K2, also known as germinal center kinase (GCK) or Rab8 interacting protein (RAB8IP), is an upstream kinase that is activated by TNF-α and is a selective activator of JNK [29]. JNK, in turn, plays an important role in mediating the cytotoxicity of various stimuli [30,31]. In addition, the role of JNK in regulating TG accumulation in the liver has been shown recently [32]. The selection of this gene as important for both TG and toxicity suggested that JNK plays an important role in these processes, which was experimentally verified (see section c below).

### Experimental validation of the identified gene-groups

#### a. Effect of treatment with fibrates on palmitate toxicity

**Figure 5:**
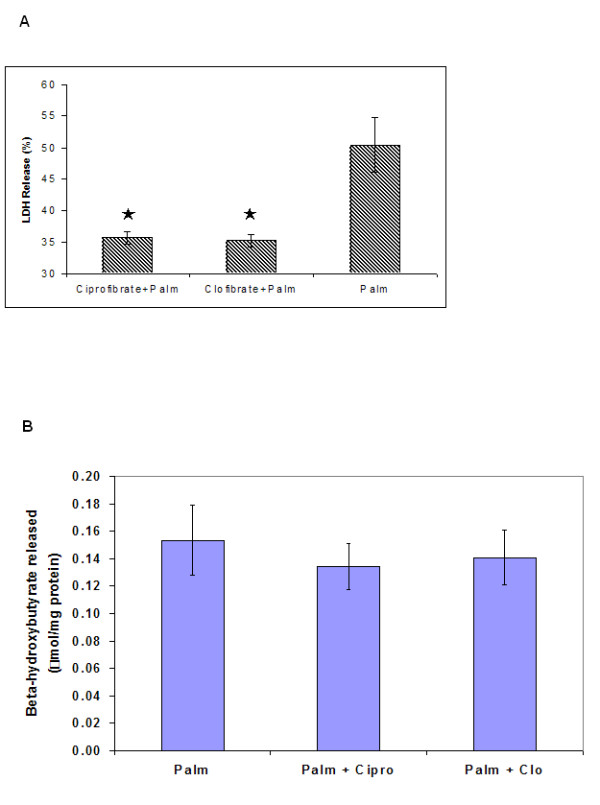
Effect of treatment with Fibrates on the cytotoxicity and ketone body release in response to palmitate. (a) Effect on cytotoxicity. Cells were pretreated with 100 uM clofibrate or ciprofibrate for 24 h, followed by treatment with 0.7 mM palmitate in the presence of 100 uM clofibrate or ciprofibrate for 48 h. The LDH released after 48 h was measured. (b) Effect on beta-hydroxybutyrate release. Cells were cultured as in (a) and the beta-hydroxybutyrate released into the medium was measured enzymatically. Data presented as mean +/- s.d. of 3 independent experiments. , significantly lower than palmitate-treated cells (p < 0.01).

The GA/PLS analysis of genes relevant to TG synthesis alone identified SCD as the most important gene. SCD has been shown to increase the synthesis of TG from palmitate and reduce the apoptosis caused by this FFA. Therefore, we tested the effect of stimulating this enzyme on the lipotoxicity. SCD was stimulated using clo- and cipro- fibrates, using a treatment regimen which has been shown to activate SCD in HepG2 cells by about 2 fold independently of the peroxisome proliferation [33]. This treatment reduced the toxicity, albeit by only about 15 % (Figure 5a). Since fibrates can also activate PPAR-α, which in turn could enhance fatty acid oxidation, we evaluated whether the reduced cytotoxicity by the fibrates was mediated by increased fatty acid oxidation. Treatment with the fibrates did not affect the ketone body release in response to palmitate (Figure 5b). This indicated that the cytoprotection due to fibrate treatments was not mediated through increased beta-oxidation.

#### b. NADH dehydrogenases

The dual-response analysis identified that NADH dehydrogenases regulated both TG accumulation and cytotoxicity. Therefore, the roles of these enzymes in these processes were investigated. NADH dehydrogenases form the mitochondrial complex I, which is involved not only in the synthesis of ATP, but also in the generation of ROS. ROS generation has been shown to be one of the important mechanisms of palmitate-toxicity [1]. We tested the effects of inhibiting NADH dehydrogenases on the cytotoxicity and lipid accumulation in response to palmitate. Inhibiting these enzymes with rotenone reduced the cytotoxicity of palmitate significantly (Table 4). Inhibiting NADH dehydrogenase reduced the toxicity by about 58%. In addition to its effect on cytotoxicity, NADH dehydrogenase also increased the accumulation of triglycerides in response to palmitate (Table 4). Inhibiting NADH dehydrogenase also increased, albeit slightly, the lipid accumulation in response to oleate (not shown). These results indicate that the NADH dehydrogenases indeed affect both lipid accumulation and cytotoxicity in response to palmitate.

**Table 4:** Effect of inhibition of NADH dehydrogenase on the cytotoxicity of palmitate and TG accumulation

**Concentration**	**LDH release (DAY 1)**	**LDH release (DAY 2)**	**TG (Day 1) ug/mg protein**	**TG (Day 2) ug/mg protein**
None	4.5 +/- 0.3	35.7 +/- 3.4	69.4 +/- 16.1	173.8 +/- 16.7
250 nM	3.4 +/- 0.3 *	24.9 +/- 1.4 *	63.3 +/- 14.2	191.1 +/- 12.1
500 nM	2.7 +/- 0.2 *	14.8 +/- 3.2 *	74.3 +/- 12.0	197.8 +/- 11.3
1000 nM	3.4 +/- 0.2 *	20.0 +/- 4.6 *	104.6 +/- 7.8 *	204.9 +/- 8.5 *

*, significantly different than the no inhibitor condition, p < 0.01.

**Figure 6:**
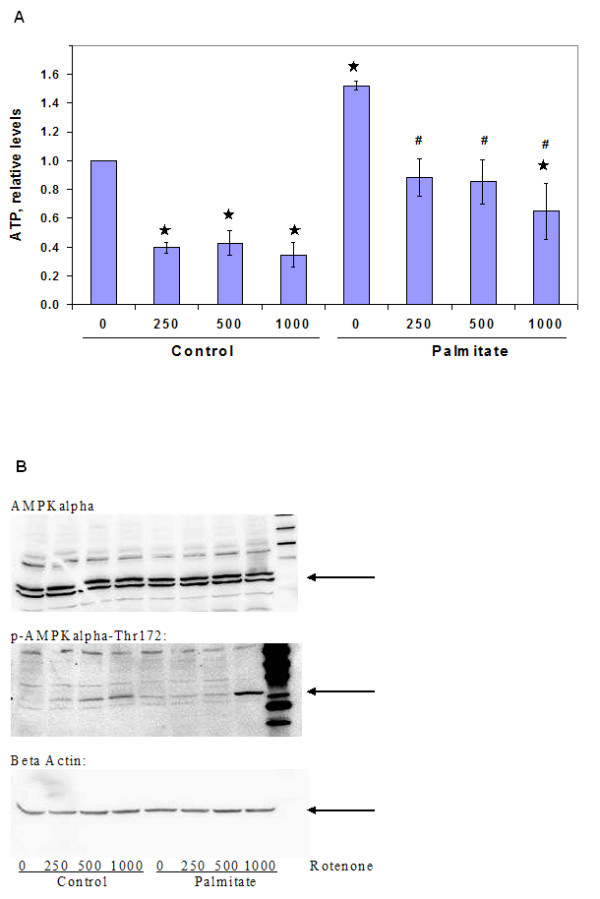
Effect of rotenone treatment. Cells were exposed to different concentrations of rotenone in control medium for 30 min, followed by treatment with either control or palmitate media for 24 h. (a) On cellular ATP levels. Cellular ATP levels were measured by luciferase assay. Data presented as mean +/- s.d. of 3 independent experiments. , significantly different from control, p < 0.01, #, significantly different from palmitate treatment, p < 0.01 (b) On AMPK activation. AMPK activation was measured through western blotting for p-Thr 172. Details of the analyses are given in the methods section.

Since the inhibition of NADH dehydrogenases could affect ATP levels in the cells, we measured ATP levels with and without rotenone in the presence and absence of palmitate. Indeed, we observed that rotenone reduced ATP levels in the presence and absence of palmitate (Figure 6a). ATP levels were higher in cells treated with palmitate and the addition of rotenone reduced ATP levels in the palmitate cultures to levels comparable to controls (Figure 6a).

Another potential mechanism by which rotenone could affect metabolism is through the activation of AMPK. We tested the effect of rotenone treatments on the activation of AMPK, measured as the AMPK phosphorylated at Thr 172. In cells cultured in control medium, there was a dose-dependent increase in AMPK activation with rotenone treatment. However, in the palmitate-treated cells, only the highest dose (1000 nM) caused an increase in AMPK activation (Figure 6b). However, the activation by rotenone in the presence of palmitate was much higher than that observed in the control cells.

#### b. MAP kinases

**Figure 7:**
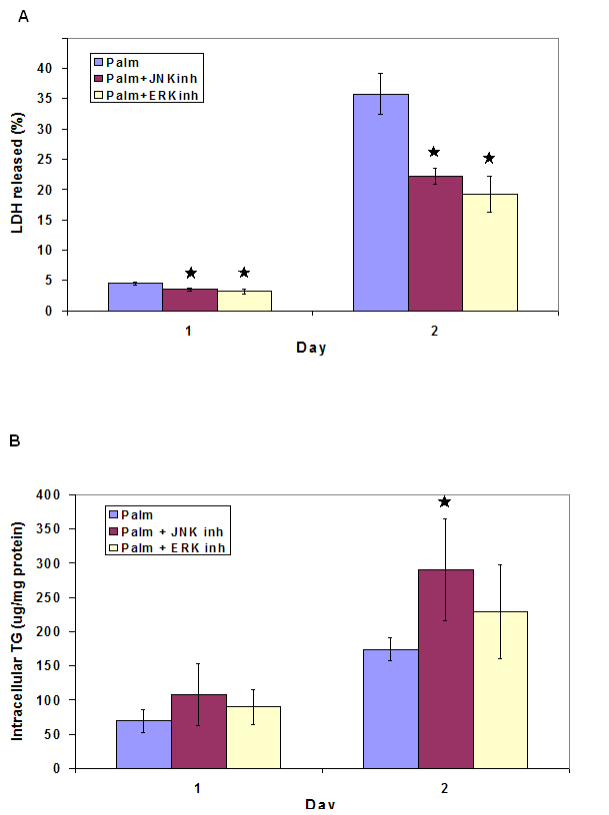
Effect of inhibition of various branches of MAPKs on the cytotoxicity and TG accumulation in response to palmitate. Cells were treated for 48 hours with palmitate in the presence of 75 uM SP-600125 (to inhibit JNK) or 50 uM U-0126 (to inhibit ERK). Media were changed daily. (a) Effect on cytotoxicity. The LDH released into the medium after 24 and 48 h of treatments was measured and normalized to the total LDH. (b) Effect on intracellular TG. Intracellular levels of TG were measured after desired time of exposure. Data presented as mean +/- s.d. of 3 independent experiments. , significantly different than palmitate (p < 0.01).

The dual response GA/PLS analysis of TG+LDH identified an important role of MAP4K2 in the regulation of these cellular responses. Because MAP4K2 is a specific upstream activator of JNK [29], this suggested an involvement of JNK in regulating both lipid accumulation and cytotoxicity. Therefore, we evaluated the effect of inhibiting JNK on the toxicity and lipid accumulation and compared the results with the effects of inhibiting other MAPK branches, i.e., ERK and p38 kinase. Indeed, inhibiting JNK reduced the toxicity significantly and increased the lipid accumulation (Figures 7a and 7b). Inhibiting ERK also reduced the toxicity significantly but did not affect the lipid accumulation (Figures 7a and 7b), while p38 had no effect on either of the two processes (not shown). Thus, among the various MAPK branches, only JNK regulate both toxicity and lipid accumulation. These results support the important roles of JNK, and therefore MAP4K2, in regulating the lipotoxicity as well as lipid accumulation as predicted by the integrative GA/PLS.

## Discussion

Hepatocytes are central to whole-body lipid metabolism. Exposure of hepatocytes to elevated FFAs and TNF-α is associated with altered lipid metabolism and cell death (lipotoxicity). Our study has identified that saturated FFA are the primary effectors of hepatic lipotoxicity and that the effect of TNF-α on the cytotoxicity are secondary to that of the saturated FFAs. We applied the dual-response GA/PLS methodology to integrate the metabolic, physiological and genetic information to identify genes relevant to discrete functions. While we have applied this analysis to only two responses of interest, the analysis is amenable to multiple quantitative responses. For complex diseases which affect multiple cellular processes, such analyses could identify targets which simultaneously regulate multiple processes and the development of complex diseases.

Our hypothesis is that the genes which can simultaneously regulate TG synthesis and lipotoxicity may be better candidates to prevent lipotoxicity than those regulating TG synthesis alone. The GA/PLS analysis for TG-alone found stearoyl-CoA desaturase (SCD) to be the most important gene regulating the synthesis of TG while the dual response analysis identified NADH dehydrogenases and JNK to be important genes for regulating both TG and LDH. We observed a decrease in SCD levels in cells treated with saturated FFA, palmitate. Although this observation is in contrast with that of others [34], it suggests a cell-specific response of SCD to fatty acids. The dichotomy of increased TG content but reduced SCD protein level could be that although SCD levels are reduced, they are not eliminated. The remaining SCD could still catalyze TG synthesis, albeit to a lesser extent. This also is supported by the observation that palmitate-treated samples had lower levels of TG accumulation as compared to oleate-treated samples. It is possible that SCD increases TG accumulation through mass action by providing more oleate and palmitoleate [35], which are better substrates for TG synthesis compared to palmitate. Secondly, the requirement of SCD for conversion of palmitate to TG is not obligatory. For example, others [36] have shown that SCD knockout mice had reduced, but not zero, incorporation of palmitate to TG. In this study, activating SCD reduced the toxicity, albeit by only about 15%, compared to a reduction of about 58% (LDH release went from 36% down to 15%) achieved by inhibiting NADH dehydrogenase or 39% by inhibiting JNK (LDH release went from 36% down to 22%). These results demonstrate greater benefits obtained by targets which regulate multiple responses.

A comparison of the genes selected for the (TG+LDH) case with those selected for TG alone showed that most (71 out of 93) of the genes selected by the dual-response analysis were not selected for the TG-alone case. Among the genes novel to the dual-response analysis were many NADH dehydrogenases genes, such as NADH dehydrogenase (ubiquinone) 1, alpha/beta subcomplex, 1, 8 kDa (NDUFAB1), NADH dehydrogenase (ubiquinone) 1 beta subcomplex, 1, 7 kDa (NDUFB1), NADH dehydrogenase (ubiquinone) 1 alpha subcomplex, 7, 14.5 kDa (NDUFA7), NADH dehydrogenase (ubiquinone) flavoprotein 2, 24 kDa (NDUFV2), NADH dehydrogenase (ubiquinone) flavoprotein 1, 51 kDa (NDUFV1). In fact, only 1 (NADH dehydrogenase (ubiquinone) 1 beta subcomplex, 4, 15 kDa) out of the 6 NADH dehydrogenase genes identified by the dual-response analysis was found in the TG-alone analysis. As our experiments demonstrated, NADH dehydrogenases played an important role in regulating TG accumulation as well as toxicity. Because NADH dehydrogenases are known to be involved in the generation of energy as well as ROS [37], their role in mediating lipotoxicity may not be very surprising. However, the GA/PLS analysis and the experiments identified that these enzymes also play an important role in regulating TG synthesis. One of the potential ways by which inhibiting NADH dehydrogenases could increase TG accumulation may be through the reduction of ATP levels. ATP has been shown to inhibit the incorporation of FFAs into TGs [38,39]. Inhibition of NADH dehydrogenases reduced ATP levels. However, the levels of ATP in the presence of palmitate and rotenone were comparable to those in control cells. This suggested that the effect of rotenone on TG was perhaps independent of the levels of ATP. Rotenone and palmitate treatment was also found to cause AMPK activation. However, previous studies have shown that AMPK activation causes increased beta-oxidation and reduced TG synthesis [40]. Therefore, the increased TG synthesis observed in cells treated with rotenone and palmitate was perhaps not mediated through AMPK activation. Rotenone could increase TG synthesis by reducing beta-oxidation, as seen in many studies [41,42]. Additionally, other mechanisms, such as increased protein kinase B (Akt1/PKB), glycogen synthase kinase 3B (GSK-3B) activation, and cAMP response element binding protein (CREB) activation, have also been shown to mediate increased TG synthesis and reduced β-oxidation in response to mitochondrial inhibition [43]. Rotenone inhibits NADH dehydrogenase and could lead to a build up of NADH in the cells. This would cause increased glycerol-1-phosphate levels [44] which could serve as a precursor for increased TG. Rotenone could also affect metabolism by reducing oxidative stress. Elevated oxidative stress could lead to reduced TG synthesis in hepatocytes [45]. However, the effects of reduced oxidative stress on TG synthesis are not clear, since another study observed reduced TG in mice fed ethanol and betaine (an anti-oxidant) as compared to mice fed ethanol without any antioxidant [46].

The MAPKs, JNK and ERK, were found to play important roles in the cytotoxicity of FFA. However, only JNK, but not ERK, affected lipid accumulation as well. It is possible that JNK inhibition may have reduced the cytotoxicity, in part, through increased TG synthesis. However, our observations of increased lipid accumulation in response to JNK inhibition are contrary to the decrease in obesity and intrahepatic lipid accumulation in the JNK-knockout mice observed in other studies [47-50]. It is likely that the differences are due to the different models (rodents in these studies vs. cell culture in ours) or the treatments (methionine choline deficient (MCD) diet in [50] vs. elevated FFA levels in ours). Identifying various mechanisms by which JNK affect lipid metabolism *in vivo *as well as *in vitro*, could shed light into the observed differences. Potential mechanisms by which JNK regulates lipid metabolism include inhibiting insulin signaling through phosphorylation of the insulin receptor at the serine residues [48] and regulating the activity of scavenger receptor element binding protein (SREBP) [51]. Likewise, the differences in the observations could also be due to the very low levels of insulin (coming only from the serum in the medium) in this study.

In designing treatment strategies for lipotoxicity, it would be useful to identify targets that reduce cell death without affecting lipid accumulation, because excessive lipid accumulation can itself alter cellular physiology and make cells prone to lipid peroxidation [52]. Our study identified ERK as another potential target to reduce the cytotoxicity without affecting lipid accumulation. While various alterations caused by FFAs have been attributed to ERK activation, such as the pro-inflammatory effects of FFAs [53], TNF-α expression [54], NF-κB expression [55], apoptosis by conjugated linoleic acid [56] and 15d-PGJ2 [57], ERK's role in fatty acid lipotoxicity has not been previously shown. The unchanged intracellular TG levels do not necessarily indicate that ERK does not affect lipid metabolism. Inhibition of ERK with U-0126 (the same inhibitor employed in this study) has been shown to improve apoB secretion by the HepG2 cells and to increase the very low density lipoprotein (VLDL) secretion in response to FFA exposure [58]. It is possible that the increased lipoprotein secretion is balanced by increased TG synthesis, that the net intracellular levels are unaffected.

Finally, our results demonstrate the applicability of the GA/PLS analysis to identify genes that regulate multiple processes of interest and suggest that such genes could be more suitable targets to regulate the development of complex diseases.

## Abbreviations (in alphabetic order)

AMPK- AMP activated protein kinase;

ANOVA- Analysis of variance;

BCA- Bicinchoninic acid;

CREB- cAMP response element binding protein;

CT- Cycle threshold;

DGAT- Diacyl glycerol acyltransferase;

DMSO- Dimethyl sulfoxide;

ERK- Extracellular signal regulated kinase;

EST- Expressed sequence tag;

FFA- Free fatty acid;

GA/PLS- Genetic algorithm coupled partial least squares;

GA- Genetic algorithm;

GCK- Germinal center kinase;

GOTM- Gene ontology tree machine;

GSK-3B- Glycogen synthase kinase 3B;

HRP- Horseradish peroxidase;

INPPL1- Inositol polyphosphatase like 1;

JNK- c-Jun N-terminal kinase;

LDH- Lactate dehydrogenase;

MAP4K2- Mitogen-activated protein kinase kinase kinase kinase 2;

MAPK- Mitogen activated protein kinase;

MGAT- Monoacyl glycerol acyltransferase;

NF-κB- Nuclear factor of kappa light polypeptide gene enhancer in B-cells;

NIPALS- Nonlinear iterative partial least squares;

PBS- Phosphate buffered saline;

PC- Principal component;

PCA- Principal component analysis;

PLS- Partial least squares;

PLS- Partial least squares;

PPAR- Peroxisome proliferator activated receptor;

PKB- Protein kinase B;

RAB8IP- Rab8 interacting protein;

ROS- Reactive oxygen species;

RT-PCR- Real time polymerase chain reaction;

SCD- Stearoyl-CoA desaturase;

SDS- Sodium dodecyl sulfate;

SHIP-2- SH2-containing inositol 5'-phosphatase 2;

SREBP- Scavenger receptor element binding protein;

TBS-T- Tris buffered saline with tween-20;

TG- Triglyceride;

TNF-α-  Tumor necrosis factor alpha;

VLDL- Very low density lipoprotein.

## Supplementary Material

Additional file 1 Determination of the number of GA/PLS runs. The data provided represent the changes in the frequency with which each gene was selected in the different number of runs. Click here for file

Additional file 2 Genes relevant to TG. This file presents a table of genes selected to be important for TG and the results of their gene ontology analysis. Click here for file

Additional file 3 Genes relevant to both TG and LDH. This file presents a table listing the genes selected to be important for both TG and LDH and the results of their gene ontology analysis. Click here for file
